# Physiological effects of bi-level high-flow nasal cannula in healthy individuals: a proof of concept trial

**DOI:** 10.3389/fmed.2025.1538832

**Published:** 2025-05-09

**Authors:** Jin Won Huh, Woo Jung Seo, Jee Hwan Ahn, Su Yeon Lee, Hee Jung Suh, Ga Jin Seo, Eun Young Kim, Min Kyung Jang, Chae-Man Lim

**Affiliations:** ^1^Department of Pulmonary and Critical Care Medicine, Asan Medical Center, University of Ulsan College of Medicine, Seoul, Republic of Korea; ^2^Division of Pulmonary and Critical Care Medicine, Department of Internal Medicine, Inje University Ilsan Paik Hospital, Inje University College of Medicine, Goyang, Republic of Korea; ^3^Respiratory Care Services, Asan Medical Center, Seoul, Republic of Korea

**Keywords:** high flow nasal cannula, Biflow, respiratory cycle, pressure-time product, concept trial

## Abstract

**Background:**

High-flow nasal cannula (HFNC) delivers a continuous, unidirectional high flow of oxygen (Uniflow) throughout the respiratory cycle. Despite its positive pressure effects in the nasopharynx, the persistent high flow during expiration imposes additional work of breathing and disrupts the patient’s neural respiratory cycle. We devised a bi-level high-flow system (Biflow) allowing separate flow rate adjustments for inspiration and expiration.

**Methods:**

We conducted a randomized crossover pilot study which we included healthy volunteer at ASAN Medical Center (April 2021 to June 2021). The data of 12 healthy volunteers (7 male, 5 female, average age 46.3 years) were analyzed. For Uniflow, flow settings of 30 (U30), 40 (U40), and 50 (U50) L/min were tested. In the Biflow, inspiratory flow rates were matched to the Uniflow settings, while expiratory flow rates varied from 10 to 30 L/min. The sequence of each flow (Uniflow vs. Biflow) was randomized and each flow setting was maintained for 3 min. Physiologic parameters, nasopharyngeal pressure-time product (N-PTP) as an energy cost proxy, end-expiratory lung impedance (EELI), and participant comfort were assessed.

**Results:**

Uniflow decreased respiratory rate and elongated expiratory time compared to natural breathing. However, these effects were less pronounced during Biflow. Compared with the Uniflow, both expiratory and inspiratory N-PTP were lower during the Biflow. Transcutaneous CO_2_ was lower during the Biflow compared with natural breathing or Uniflow. EELI did not differ between modes. All participants completed the study protocol without side effects.

**Conclusion:**

In healthy participants, compared with the conventional HFNC (Uniflow), Biflow showed less interference with the natural respiratory cycle of the participants. Compared with Uniflow, energy cost occurring in the nasopharynx was lower during Biflow

**Clinical trial registration:**

http://cris.nih.go.kr/cris/, identifier KCT0006100.

## Introduction

High-flow nasal cannula (HFNC) is widely employed in patients with hypoxemia and moderate hypercapnia (1–5). By maintaining a continuous high flow during expiration, HFNC generates a low-to-moderate level of positive end-expiratory pressure (PEEP) in the nasopharynx (6–14). This mechanism enhances lung volume and improves oxygenation (6, 7, 15, 16). The high inspiratory flow provided by HFNC reduces inspiratory work of breathing (WOB). However, during expiration, patients must overcome the resistance generated by the incoming high flow, potentially leading to increased WOB and disruption of the neural respiratory cycle (11, 17). We have therefore developed a bi-level high-flow system (Biflow), allowing separate adjustment rates for inspiration and expiration.

In the Biflow system, for instance, the inspiratory flow rate is set above the needs of the participants (e.g., 40 L/min), while the expiratory flow rate is set lower than the inspiratory flow rate (e.g., 20 L/min) to facilitate the participants’ own expiration. Our study aimed to explore the physiologic effects of Biflow in healthy individuals and compare them with those of conventional HFNC (Uniflow).

## Materials and methods

This study was conducted at Asan Medical Center in Seoul, South Korea, a university-affiliated hospital, from April 2021 to June 2021. The study was approved by the Institutional Review Board (IRB), and informed consent was obtained from each participant (IRB No-2020-1901; approval date: December 23, 2020). The registration of the clinical trial was made prior to the enrollment of the first patient. The procedures were followed in accordance with the ethical standards of the responsible committee of ASAN Medical Center on human experimentation and the Helsinki Declaration of 1975.

### Eligible individuals

This study was a proof of concept trial for Biflow in healthy individuals to evaluate the physiologic effects of the Biflow system. Inclusion criteria encompassed healthy adults aged 18 years or older with no history of respiratory or cardiovascular disease. Exclusion criteria included conditions precluding the application of a nasal respiratory cannula, such as previous facial surgery, trauma, deformity, or airway obstruction.

### Intervention

**FIGURE 1:**
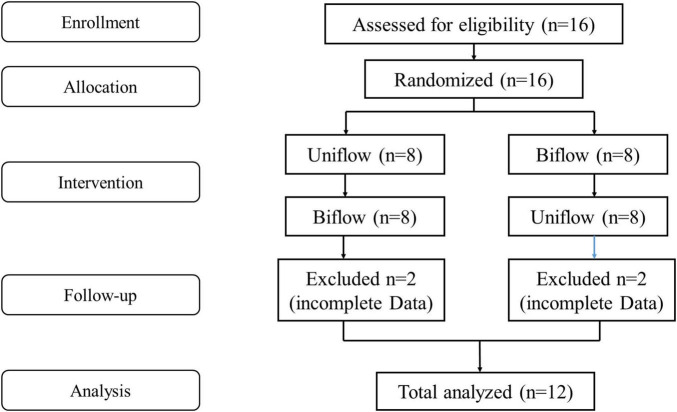
Consort flow diagram.

This prospective, controlled study employed a randomized block design mixing block sizes of 4 and 6 in healthy volunteers, initiating either Uniflow or Biflow mode (Figure 1).

**FIGURE 2:**
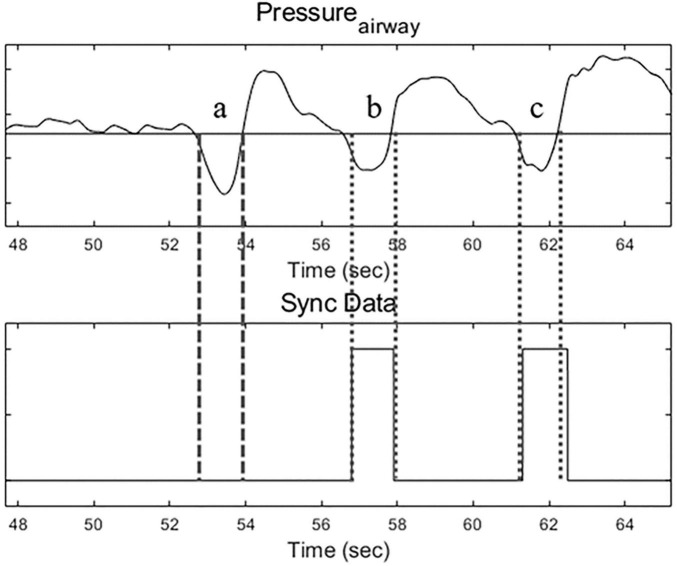
Airway pressure tracing (upper) and Bi-flow operating signal (lower). During Biflow, breath a was a failed operation, and breaths b and c were successful.

For the Uniflow mode, three flow rates were examined: 30 L/min (U30), 40 L/min (U40) and 50 L/min (U50). In the Biflow mode, inspiratory flow rates were aligned with those of Uniflow, while expiratory (basal) flow rates varied: 10, 20, and 30 L/min. The resultant flow settings for Biflow were (inspiratory/expiratory): 30/10, 30/20, 40/10, 40/20, 40/30, 50/20, 50/30. Biflow 50/40 was excluded as most volunteers exhibited intolerance during the preliminary test. The phase transition between expiration and inspiration in the Biflow system relied on a “patient effort-dependent trigger.” The criteria for the initiation of inspiration were met when the nasopharyngeal pressure dropped by ≥ 0.4 cmH_2_O, accompanied by a simultaneous increase in flow rate by ≥ 1 L/min. Alternatively, expiration was triggered if the inspiratory phase exceeded the predefined time limit (6 s). However, the current version of the Biflow system failed to detect the inspiratory effort of the patient under conditions of relatively low bias flow (before the start of inspiration) or when the participant’s respiratory effort was too weak. Owing to this technical constraint, the proportion of breaths delivered through the Biflow system ranged between 25 and 63% of all recorded breaths (Figure 2). The sequence and duration of each flow setting were randomized and maintained for 3 min. Baseline recordings of spontaneous breathing were recorded for 5 mins before beginning the study protocol. Between Uniflow and Biflow, a 5-min washout period was allowed for the resumption of normal breathing (18).

### Measurements

Physiologic parameters, including respiratory rates (RR), inspiratory:expiratory (I:E) ratio, heart rate (HR), transcutaneous CO_2_ (tcCO_2_) and pulse oxygen saturation (SpO_2_), were systematically recorded at each minute of the study. Subjective comfort levels were evaluated using the modified Borg Scale (MBS) at the end of each setting. To compare end-expiratory lung volume, continuous electrical impedance tomography (EIT) data were collected throughout the study (19–21). EIT generates cross-sectional images depicting impedance distribution within electrically conductive objects, allowing for a semi-quantitative evaluation of static and dynamic lung volumes. Tidal impedance variation (TIV) and global inhomogeneity (GI) index were analyzed using EIT Data Analysis Tool version 6.3 and GI MATLAB Tool ver 3.0. End-expiratory lung impedance (EELI) data were processed using dedicated software Pulmovista Data Analysis Software ver 1.3.

To gauge the nasopharyngeal pressure of the participant, a thin PVC catheter (7 Fr) was inserted 9 cm deep in one of the nostrils of the participant. The pressure signal was recorded using LabVIEW (National Instruments Co., TX, United States), and subsequent numerical analyses were executed using MATLAB software (Mathworks Inc., MA, United States). ΔPexp (cmH_2_O) was defined as (peak pressure – baseline pressure) during expiration, and ΔPinsp (cmH_2_O) as (baseline pressure – lowest pressure) during inspiration. The nasal pressure-time produced (N-PTP), a proxy of energy cost (22, 23), was calculated by integrating *P*_*aw*_ with time: ∫(*P*_*aw*_−*PEEP*)*dt*. Inspiratory, expiratory, and total N-PTP were obtained (Supplementary Figure 1) (24, 25). Inspiration time and expiration time were also measured.

### Statistical analysis

All continuous variables are presented as median and interquartile range, while categorical variables are expressed as percentages. Wilcoxon signed-rank test was employed for continuous data, and Pearson’s chi-squared test or Fisher’s exact test for categorical data. Comparisons between Uniflow and Biflow parameters at the same inspiratory flow rate were conducted, employing pooled and matched analyses. All *p*-values were two-tailed, with statistical significance set at *p* < 0.05. All statistical analyses were performed using SPSS software (version 22.0; IBM Corporation, Somers, NY, United States).

## Results

We enrolled sixteen healthy participants, of whom all followed the protocol until completion. Among them, 12 cases acquired the full measurement data and were included in the analysis. The participant population comprised 58% males, and the mean age was 46.2 years. The median BMI was 22.1 (19.7–25.5) kg/m^2^.

As the flow rate increased, RR decreased significantly during Uniflow settings compared with natural breathing; however, RR during the Biflow settings did not differ from the natural breathing. During the Uniflow settings, both inspiratory and expiratory times were prolonged compared with natural breathing. As the flow rate was increased during the Uniflow, the expiratory time was more elongated. During the Biflow settings, inspiratory time was prolonged, but expiratory time did not significantly differ from natural breathing. Expiratory time was shorter with the Biflow compared with Uniflow. The I:E ratio remained unchanged during Uniflow but increased during Biflow as the flow rate increased (Table 1).

**TABLE 1:** Change of synchronized respiratory parameters according to the flow setting.

	Setting	RR (/min)	Inspiratory time (sec)	Expiratory time (sec)	I:E ratio
Baseline	Natural breathing	15.6 (13.5–17.4)	1.3 (1.1–1.4)	2.3 (1.8–2.7)	0.5 (0.5–0.6)
Flow 30	U30	13.5 (10.0–15.8)*	1.7 (1.5–2.2)*	2.9 (2.1–4.0)*	0.7 (0.5–0.8)*
	B30/10	13.3 (12.0–18.0)	1.9 (1.6–2.2)*	2.4 (1.9–2.6)	0.8 (0.7–1.0)*^,^**
	B30/20	13.9 (13.1–17.9)**	1.6 (1.3–1.8)*	2.3 (1.9–2.8)**	0.7 (0.6–0.9)*
Flow 40	U40	12.7 (9.1–13.8)*	1.7 (1.5–2.1)*	3.1 (2.7–4.8)*	0.5 (0.5–0.6)
	B40/10	13.0 (12.3–14.7)**	1.9 (1.7–2.5)*	2.5 (2.2–3.0)**	0.7 (0.7–0.9)*^,^**
	B40/20	16.4 (12.7–18.2)**	1.5 (1.1–2.1)^†^	2.2 (1.9–2.9)**	0.6 (0.5–0.8)
	B40/30	13.7 (10.5–14.2)**^,^^‡^	1.6 (1.5–1.9)*^,^^†^	3.0 (2.7–4.5)**^,^^‡^	0.6 (0.5–0.6)**^,^^†^
Flow 50	U50	10.0 (9.4–13.2)*	1.8 (1.6–2.0)*	3.8 (2.7–4.5)*	0.5(0.4–0.6)
	B50/20	15.0 (12.9–19.8)**	1.7 (1.3–2.2)*	2.4 (1.9–3.1)**	0.7 (0.6–0.8)*^,^**
	B50/30	14.2 (11.0–16.6)**^,^^‡^	1.7 (1.5–2.2)*^,^^‡^	2.5 (2.0–2.9)**	0.7 (0.6–0.9)*^,^**

Data are expressed as median and IQR.

*P-value of < 0.05 compared with natural breathing,

**P-value of < 0.05 compared with Uniflow.

^†^*P*-value of < 0.05 compared with Biflow 40/10,

^‡^*P*-value of < 0.05 compared with Biflow 40/20 or 50/20.

No significant differences were observed in HR and SpO_2_ between the Unflow and the Biflow at the same inspiratory flow rate. The levels of tcCO_2_ at B30/10, B40/10, and B40/20 were notably lower compared with natural breathing or the Uniflow settings (Table 2). The participants reported light dyspnea during both modes.

**TABLE 2:** Change in physiological variables according to the flow setting.

	Setting	HR (/min)	tcCO_2_ (mmHg)	SpO_2_ (%)	MBS
Baseline	Natural breathing	72.0 (68.6–85.8)	38.8 (35.6–41.2)	97.8 (96.9–98.7)	0
Flow 30	U30	67.8 (65.9–84.8)*	39.5 (36.1–41.9)	97.7 (97.2–98.8)	0 (0–0.5)*
	B30/10	68.9 (65.9–87.0)	37.4 (34.2–41.0)**	98.0 (97.2–99.0)	0.4 (0–0.9)*
	B30/20	68.2 (66.6–86.6)	38.3 (35.0–41.2)^†^	98.0 (97.2–99.)	0.5 (0–0.5)*
Flow 40	U40	70.9 (66.6–86.4)	38.2 (36.1–40.2)	98.0 (97.4–98.8)	0.5 (0–0.5)*
	B40/10	69.7 (66.3–86.0)	37.0 (34.2–40.5)*^,^**	98.0 (97.0–99.0)	0.5 (0–2.0)*
	B40/20	69.5 (67.0–84.7)*	37.7 (33.2–40.6)*^,^**	98.0 (97.2–99.0)	0.3 (0–1.8)*
	B40/30	69.7 (67.1–82.3)*	39.2 (35.9–40.8)^†^	98.2 (97.0–99.3)	0.3 (0–1.6)*
Flow 50	U50	71.0 (68.0–87.7)	37.9 (36.0–40.2)	98.0 (96.9–98.7)	0.8 (0.5–1.8)*
	B50/20	68.8 (66.1–82.2)*	37.1 (33.6–39.9)*	98.0 (96.9–98.7)	0.5 (0.1–2.0)*
	B50/30	71.7 (67.7–82.2)	36.6 (33.9–39.8)*	98.0 (96.9–98.7)	0.3 (0–1.6)*

Data are expressed as median and IQR.

*P-value of < 0.05 compared with natural breathing,

**P-value of < 0.05 compared with Uniflow.

^†^*P*-value of < 0.05 compared with Biflow (30/10 or 40/10).

### Nasal pressure-time product

**FIGURE 3:**
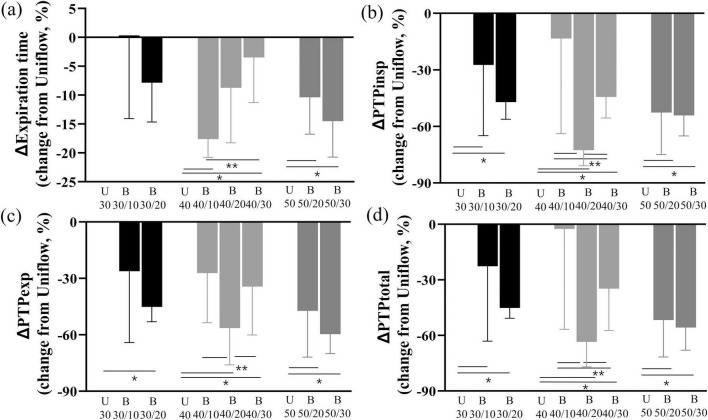
Changes in expiratory time and N-PTP **(a)** Change of expiration time of Biflow compared to Uniflow **(b)** Change of inspiratory PTP of Biflow compared to Uniflow **(c)** Change of expiratory PTP of Biflow compared to Uniflow **(d)** Change of total PTP of Biflow compared to Uniflow *P*-value of <0.05 compared with Uniflow, *P-value of <0.05 between Biflow settings. PTPinsp, inspiratory nasal pressure time product; PTPexp, expiratory nasal pressure time product; PTPtotal, total nasal pressure time product; U, Uniflow; B, Biflow.

Expiratory time was shorter during the Biflow than during the Uniflow (Table 1 and Figure 3a). Changes in the nasopharyngeal pressures during the Biflow as compared with the Uniflow are presented in Supplementary Table 1. Inspiratory N-PTP (Figure 3b) and expiratory N-PTP (Figure 3c) were lower during Biflow compared with Uniflow. Total N-PTP was also lower during Biflow than Uniflow (Figure 3d). The decrease in N-PTP was most prominent at B40/20 (inspiratory flow 40 L/min and expiratory flow 20 L/min).

### Electrical impedance tomography measurements

**FIGURE 4:**
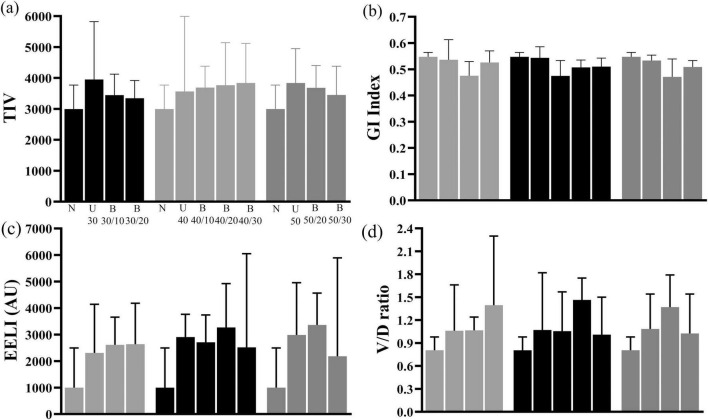
Changes in TIV, GI index, EELI, and V/D ratio of EELI **(a)** Comparison of TIV among natural breathing, Uniflow and Biflow settings **(b)** Comparison of GI Index among natural breathing, Uniflow and Biflow settings **(c)** Comparison of EELI among natural breathing, Uniflow and Biflow settings **(d)** Comparison of V/D ratio among natural breathing, Uniflow and Biflow settings *P*-value of <0.05 compared with natural breathing, *P-value of <0.05 between Biflow settings. TIV, tidal impedance variation; GI, global inhomogeneity; EELI, end-expiratory lung impedance; V/D ratio, ventral/dorsal ratio; U, Uniflow; B, Biflow.

TIV, EELI and V/D ratio increased in both Uniflow and Biflow modes compared with natural breathing. However, there were no significant differences observed between the two modes (Figure 4). GI index exhibited a decrease in B40/10, B40/20, and B50/20 compared with natural breathing.

## Discussion

This study was designed as an exploratory trial to evaluate the Biflow system in healthy individuals. Compared with conventional HFNC (Uniflow), Biflow demonstrated a less substantial decrease in respiratory rate. During Uniflow, as the flow rate increased, the respiratory rate exhibited a stepwise decrease compared with natural breathing, while both inspiratory and expiratory time were elongated. However, during Biflow, the decrease in respiratory rate was less pronounced, and the expiratory time was preserved, resembling the pattern observed in the natural breathing of the participants. Compared with Uniflow, the N-PTP during both inspiration and expiration was lower with Biflow. These findings suggest that the energy dissipated in the nasopharynx during breathing was reduced with the Biflow compared with Uniflow. Interestingly, the lower expiratory N-PTP during Biflow was attributed to the decreased expiratory time compared with Uniflow, per the N-PTP formula (integration of pressure change and time). Conversely, the lower inspiratory N-PTP during Biflow was attributed to an attenuated pressure change during the inspiratory phase compared with Uniflow. Physiologic data such as HR, BP, SpO_2_ and EELI, were similar between the two HFNC systems in the participants. The tcCO_2_ levels during Biflow at flow trials 30 and 40 were lower compared with natural breathing or Uniflow. We observed a slight decrease in heart rate during both Uniflow and Biflow compared to natural breathing. This finding likely reflects reduced work of breathing due to high-flow oxygen support (26) or changes in vagal tone resulting from comfortable breathing pattern (27). The changes in the heart rate during both Uniflow and Biflow were modest (approximately 2-3 beats/min) and were similar between the two modes.

The use of HFNC, referred to as Uniflow in our study, has increased exponentially over the past few years, particularly during the COVID-19 pandemic (1). The adoption of HFNC helped reduce intubation rates for patients with acute hypoxic respiratory failure and prevent post-extubation respiratory failure (2, 3). Improved patient outcomes associated with HFNC are due to physiological effects, such as improvement in oxygenation (15, 28), efficient ventilation (15, 29), avoidance of patient self-inflicted lung injury, and improvement in patient comfort and tolerance (30). For maximal dead space washout and PEEP-like effect, clinical use of the flow rate during HFNC ranges up to 40–50 L/min. Dysart et al. suggested that matching HFNC with inspiratory demand may attenuate nasopharyngeal resistance and lead to a reduction of WOB. Despite these benefits, this excessive range of gas flow may impose additional WOB to overcome the greater jet flow for expiration (31) and disrupt patients’ own respiratory cycle. HFNC has been widely reported to affect respiratory rate, tidal volume, and I:E ratio (11, 32, 33). Our study also showed that HFNC led to a decrease in respiratory rate and an increase in inspiratory time and expiratory time compared with natural breathing. However, the Biflow system, characterized by alternating flow rates synchronized with the respiratory cycle—specifically, a reduction of flow during the expiratory phase—mitigated the bradypnea effect associated with high flow and increased the I:E ratio. Notably, inspiratory pressure changes in the nasopharynx during the Bioflow were attenuated and inspiratory N-PTP was consequently lower compared with the Uniflow. The combination of reduced pressure changes and increased I:E ratio, particularly with a reduced expiration time with the Biflow mode, suggests a potential reduction in the energy cost per breathing cycle.

As the PEEP-like effect during HFNC is known to be proportional to flow rate (18), the lower flow rate during expiration with the Biflow may be disadvantageous in terms of the PEEP-like effect or end-expiratrory lung volume. In our study, the expiratory nasopharyngeal pressure during the Biflow was lower than Uniflow by 27.2-83.7 % (Supplementary Table 1). Whereas, the EELI (surrogate for volume) as assessed by EIT was not different between Uniflow and Biflow (Figure 4). It seems, therefore, premature to predict the overall effect of the reduced expiratory nasopharyngeal pressure during Biflow in diverse respiratory patients.

Besides oxygenation aid, the HFNC is used as a ventilation support for mild-moderate hypercapnia (4, 5). In the Biflow system, the augmentation of gas flow during inspiration is analogous to the pressure augmentation occurring with BiPAP, a noninvasive ventilation method. BiPAP necessitates a mask or helmet to create a closed circuit, which is often the culprit of treatment failure. Unlike BiPAP, Biflow operates without the need for these artificial interfaces. In this context, Biflow may be regarded as an ‘open’ system for noninvasive ventilation support, although the level of support may be lower than with BiPAP.

Our study has a few limitations. First, this is a single-center study with a small number (twelve) of participants. However, as this was the first study examining the physiological effects of the Biflow, we determined the sample size based on existing studies of HFNC. Studies by Ritchie et al. (34), Parke et al. (18), and Groves and Tobin (35) used 10-15 subjects to examine various physiological parameters of HFNC. Second, breath synchronization during the Biflow system was not technically perfect. For the operation of the Biflow, both inspiratory and expiratory efforts of the participant should be detected. The current Biflow system failed to detect the inspiration of the participant when the bias flow rate (before the start of inspiration) was relatively low (e.g., 10 L/min) or when the breathing effort was too weak. This technical limitation is inherently associated with the open nature of the Biflow system and requires improvement before clinical application. Finally, we did not investigate the long-term effects of the Biflow. Prior to establishing any clinical benefit, the Biflow needs long-term application in patients with various respiratory pathophysiologies.

## Conclusion

Compared with the current HFNC (singular flow throughout the respiratory cycle), the Biflow HFNC in healthy individuals showed less disturbance of participants’ respiratory cycle and resulted in a lower energy cost occurring in the nasopharynx.

## Data Availability

The original contributions presented in this study are included in this article/Supplementary material, further inquiries can be directed to the corresponding author.

## References

[1] Papoutsi E Giannakoulis V Xourgia E Routsi C Kotanidou A Siempos I. Effect of timing of intubation on clinical outcomes of critically ill patients with COVID-19: A systematic review and meta-analysis of non-randomized cohort studies. *Crit Care.* (2021) 25:121. 10.1186/s13054-021-03540-6 33766109 PMC7993905

[2] Rochwerg B Granton D Wang D Helviz Y Einav S Frat J High flow nasal cannula compared with conventional oxygen therapy for acute hypoxemic respiratory failure: A systematic review and meta-analysis. *Intensive Care Med.* (2019) 45:563–72. 10.1007/s00134-019-05590-5 30888444

[3] Fernando S Tran A Sadeghirad B Burns K Fan E Brodie D Noninvasive respiratory support following extubation in critically ill adults: A systematic review and network meta-analysis. *Intensive Care Med.* (2022) 48:137–47. 10.1007/s00134-021-06581-1 34825256

[4] Feng Z Zhang L Yu H Su X Shuai T Zhu L High-flow nasal cannula oxygen therapy versus non-invasive ventilation for AECOPD patients after extubation: A systematic review and meta-analysis of randomized controlled trials. *Int J Chron Obstruct Pulmon Dis.* (2022) 17:1987–99. 10.2147/COPD.S375107 36065316 PMC9440713

[5] Tan D Walline J Ling B Xu Y Sun J Wang B High-flow nasal cannula oxygen therapy versus non-invasive ventilation for chronic obstructive pulmonary disease patients after extubation: A multicenter, randomized controlled trial. *Crit Care.* (2020) 24:489. 10.1186/s13054-020-03214-9 32762701 PMC7407427

[6] Chikata Y Onodera M Oto J Nishimura M. FIO2 in an adult model simulating high-flow nasal cannula therapy. *Respir Care.* (2017) 62:193–8. 10.4187/respcare.04963 27879385

[7] Sun Y Dai B Peng Y Tan W Zhao H. Factors affecting FiO2 and PEEP during high-flow nasal cannula oxygen therapy: A bench study. *Clin Respir J.* (2019) 13:758–64. 10.1111/crj.13087 31465634

[8] Hebbink R Duiverman M Wijkstra P Hagmeijer R. Upper airway pressure distribution during nasal high-flow therapy. *Med Eng Phys.* (2022) 104:103805. 10.1016/j.medengphy.2022.103805 35641081

[9] Luo J Lu M Zhao Z Jiang W Xu B Weng L Positive end-expiratory pressure effect of 3 high-flow nasal cannula devices. *Respir Care.* (2017) 62:888–95. 10.4187/respcare.05337 28442633

[10] Nielsen K Ellington L Gray A Stanberry L Smith L DiBlasi R. Effect of high-flow nasal cannula on expiratory pressure and ventilation in infant, pediatric, and adult models. *Respir Care.* (2018) 63:147–57. 10.4187/respcare.05728 29066588

[11] Adams C Geoghegan P Spence C Jermy M. Modelling nasal high flow therapy effects on upper airway resistance and resistive work of breathing. *Respir Physiol Neurobiol.* (2018) 254:23–9. 10.1016/j.resp.2018.03.014 29635072

[12] Onodera Y Akimoto R Suzuki H Okada M Nakane M Kawamae K. A high-flow nasal cannula system with relatively low flow effectively washes out CO2 from the anatomical dead space in a sophisticated respiratory model made by a 3D printer. *Intensive Care Med Exp.* (2018) 6:7. 10.1186/s40635-018-0172-7 29546563 PMC5854566

[13] Moore C Katz I Pichelin M Caillibotte G Finlay W Martin A. High flow nasal cannula: Influence of gas type and flow rate on airway pressure and CO2 clearance in adult nasal airway replicas. *Clin Biomech.* (2019) 65:73–80. 10.1016/j.clinbiomech.2019.04.004 30991233

[14] Guérin C Cour M Degivry F Argaud L Louis BA. Bench comparison of the effect of high-flow oxygen devices on work of breathing. *Respir Care.* (2022) 67:1129–37. 10.4187/respcare.09889 35790397

[15] Li J Scott J Fink J Reed B Roca O Dhand R. Optimizing high-flow nasal cannula flow settings in adult hypoxemic patients based on peak inspiratory flow during tidal breathing. *Ann Intensive Care.* (2021) 11:164. 10.1186/s13613-021-00949-8 34837553 PMC8626729

[16] Duprez F de Terwangne C Bellemans V Poncin W Reychler G Sorgente A High-flow nasal cannula therapy, factors affecting effective inspired oxygen fraction: An experimental adult bench model. *J Clin Monit Comput.* (2022) 36:1441–8. 10.1007/s10877-021-00784-z 34877626 PMC8651462

[17] Chang G Cox C Shaffer T. Nasal cannula, CPAP, and high-flow nasal cannula: Effect of flow on temperature, humidity, pressure, and resistance. *Biomed Instrum Technol.* (2011) 45:69–74. 10.2345/0899-8205-45.1.69 21322815

[18] Parke R McGuinness S Eccleston M. Nasal high-flow therapy delivers low level positive airway pressure. *Br J Anaesth.* (2009) 103:886–90. 10.1093/bja/aep280 19846404 PMC2777940

[19] Frerichs I. Electrical impedance tomography (EIT) in applications related to lung and ventilation: A review of experimental and clinical activities. *Physiol Meas.* (2000) 21:R1–21. 10.1088/0967-3334/21/2/201 10847187

[20] Pulletz S van Genderingen H Schmitz G Zick G Schädler D Scholz J Comparison of different methods to define regions of interest for evaluation of regional lung ventilation by EIT. *Physiol Meas.* (2006) 27:S115–27. 10.1088/0967-3334/27/5/S10 16636403

[21] Zhao Z Yun P Kuo Y Fu F Dai M Frerichs I Comparison of different functional EIT approaches to quantify tidal ventilation distribution. *Physiol Meas.* (2018) 39:01NT01. 10.1088/1361-6579/aa9eb4 29192891

[22] Calzia E Lindner K Witt S Schirmer U Lange H Stenz R Pressure-time product and work of breathing during biphasic continuous positive airway pressure and assisted spontaneous breathing. *Am J Respir Crit Care Med.* (1994) 150:904–10. 10.1164/ajrccm.150.4.7921461 7921461

[23] Montecchia F Luciani S Cicchetti R Grossi R Midulla F Moretti C Pharyngeal and esophageal pressure measurements to evaluate respiratory mechanics in infants on high flow nasal cannula: A feasibility study. *Proceedings of the IEEE International Symposium on Medical Measurements and Applications (MeMeA).* Turin: IEEE (2015).

[24] Grinnan D Truwit J. Clinical review: Respiratory mechanics in spontaneous and assisted ventilation. *Crit Care.* (2005) 9:472–84. 10.1186/cc3516 16277736 PMC1297597

[25] Natalini G Marchesini M Tessadrelli A Rosano A Candiani A Bernardini A. Effect of breathing pattern on the pressure-time product calculation. *Acta Anaesthesiol Scand.* (2004) 48:642–7. 10.1111/j.0001-5172.2004.00377.x 15101863

[26] Li J Albuainain F Tan W Scott J Roca O Mauri T. The effects of flow settings during high-flow nasal cannula support for adult subjects: A systematic review. *Crit Care.* (2023) 27:78. 10.1186/s13054-023-04361-5 36855198 PMC9974062

[27] Lehrer P Gevirtz R. Heart rate variability biofeedback: How and why does it work? *Front Psychol.* (2014) 5:756. 10.3389/fpsyg.2014.00756 25101026 PMC4104929

[28] Mauri T Alban L Turrini C Cambiaghi B Carlesso E Taccone P Optimum support by high-flow nasal cannula in acute hypoxemic respiratory failure: Effects of increasing flow rates. *Intensive Care Med.* (2017) 43:1453–63. 10.1007/s00134-017-4890-1 28762180

[29] Delorme M Bouchard P Simon M Simard S Lellouche F. Effects of high-flow nasal cannula on the work of breathing in patients recovering from acute respiratory failure. *Crit Care Med.* (2017) 45:1981–8. 10.1097/CCM.0000000000002693 28857852

[30] Goligher E Slutsky A. Not just oxygen? Mechanisms of benefit from high-flow nasal cannula in hypoxemic respiratory failure. *Am J Respir Crit Care Med.* (2017) 195:1128–31. 10.1164/rccm.201701-0006ED 28459344

[31] Dysart K Miller T Wolfson M Shaffer T. Research in high flow therapy: Mechanisms of action. *Respir Med.* (2009) 103:1400–5. 10.1016/j.rmed.2009.04.007 19467849

[32] Bräunlich J Beyer D Mai D Hammerschmidt S Seyfarth H Wirtz H. Effects of nasal high flow on ventilation in volunteers, COPD and idiopathic pulmonary fibrosis patients. *Respiration.* (2013) 85:319–25. 10.1159/000342027 23128844

[33] Corley A Caruana L Barnett A Tronstad O Fraser J. Oxygen delivery through high-flow nasal cannulae increase end-expiratory lung volume and reduce respiratory rate in post-cardiac surgical patients. *Br J Anaesth.* (2011) 107:998–1004. 10.1093/bja/aer265 21908497

[34] Ritchie J Williams A Gerard C Hockey H. Evaluation of a humidified nasal high-flow oxygen system, using oxygraphy, capnography and measurement of upper airway pressures. *Anaesth Intensive Care.* (2011) 39:1103–10. 10.1177/0310057X1103900620 22165366

[35] Groves N Tobin A. High flow nasal oxygen generates positive airway pressure in adult volunteers. *Aust Crit Care.* (2007) 20:126–31. 10.1016/j.aucc.2007.08.001 17931878

