# Supplementary material for: Physiological effects of bi-level high-flow nasal cannula in healthy individuals: a proof of concept trial
Source: Front Med (Lausanne). 2025 May 9;12:1538832. doi: 10.3389/fmed.2025.1538832 (PMC12098088; doi:10.3389/fmed.2025.1538832)
Supplement: Supplementary file 1 [file Data_Sheet_1.pdf]

Supplement Figure 1. Area a is inspiratory N-PTP, and area b is expiratory N-PTP.

Total N-PTP is the sum of a and b. The values of N-PTP are the average values taken of the breaths during the middle 1 minute during the 3-minute period.

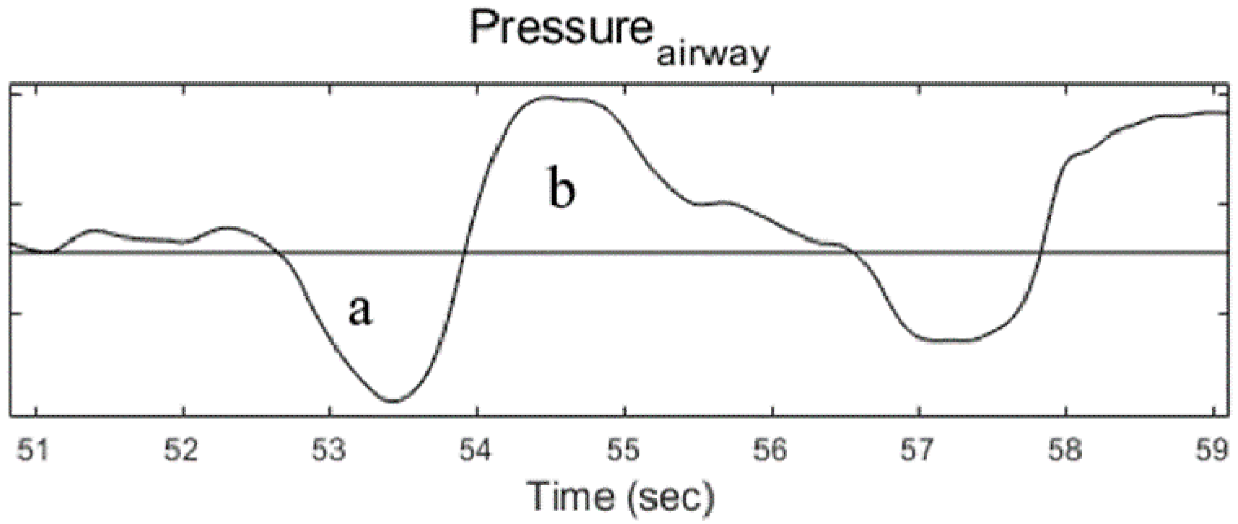

Supplement table 1. Changes of Pinsp and Pexp

	Setting	Pinsp, %	Pexp, %
Flow 30	U30	0	0
	B30/10	-27.4 (-64.9–10.0)*	-35.6 (-182.2–0.0)
	B30/20	-47.2 (-56.2–-41.1)*	-83.7(-113.0–-36.9)*
Flow40	U40	0	0
	B40/10	-13.4 (-63.8–53.9)	-27.2 (-53.6–17.2)
	B40/20	-72.6 (-80.9–-41.1)*,**	-56.4 (-76.0–-48.5)*,**
	B40/30	-44.4 (-55.5–-26.2)*,**,†	-34.4 (-60.1–-14.1)*,†
Flow50	U50	0	0
	B50/20	-52.6 (-74.9–-32.6)*	-47.3 (-71.9–-19.5)*
	B50/30	-54.2 (-65.1–-21.0)*	-59.7 (-70.0–-12.4)*

Data are expressed as median and IQR, *P-value of < 0.05 compared with Uniflow, **P-value of < 0.05 compared with Biflow 40/10, † P-value of < 0.05 compared with Biflow 40/20.
